# Does Remote Monitoring Improve Time on Peritoneal Dialysis?: CON

**DOI:** 10.34067/KID.0000001046

**Published:** 2025-12-05

**Authors:** Abdullah Al Thani, Arsh K. Jain, Jeffrey Perl

**Affiliations:** 1 Department of Medicine, King Abdulaziz Hospital, Ministry of National Guard Health Affairs, Hofuf, Saudi Arabia; 2 Division of Nephrology, London Health Sciences Centre and Western University, London, Ontario, Canada; 3 Division of Nephrology, University of Toronto, Toronto, Ontario, Canada; 4 Division of Nephrology, St. Michael's Hospital, University of Toronto, Toronto, Ontario, Canada

**Keywords:** dialysis, peritoneal dialysis

Remote patient monitoring (RPM) refers to the use of digital technologies to collect and transmit health data from patients in their homes to health care providers, most commonly including biometric measures, such as weight and BP. As a form of telehealth, RPM seeks to improve clinical outcomes by facilitating earlier diagnosis, enabling timely intervention, preventing complications, and ultimately reducing hospitalizations and mortality. It also aims to enhance patient-reported outcomes, such as treatment satisfaction and quality of life. In the context of peritoneal dialysis (PD), an important outcome is the prevention of premature discontinuation and subsequent transition to another dialysis modality—a priority highlighted by the international Standardized Outcomes in Nephrology–Peritoneal Dialysis (SONG-PD) initiative.^1^ Although much of the focus of RPM technology in PD has been on connected automated peritoneal dialysis (APD) cyclers, which record and transmit detailed treatment data over the Internet such as session completion, inflow and outflow volumes, and machine alarms, RPM more broadly encompasses any digital platform that relays treatment and biometric information. This provides clinicians with a comprehensive, near real-time view of patients' health status, offering the potential to improve safety, support adherence, and sustain long-term use of PD.

Does RPM in its current form prevent premature discontinuation of PD and the subsequent transition to an alternate dialysis modality? Preventing this transition has been identified as a key priority as a core outcome for clinical trials developed by a diverse group of patients and PD care stakeholders in the SONG-PD initiative.^1^ In PD, RPM and its related research have become closely associated with connected APD cyclers, but RPM encompasses all transmitted health information *via* dedicated digital platforms. These systems capture and transmit daily treatment information, including data on completed treatments, fluid inflow and outflow parameters, and machine alarms, alongside key biometric information.

In PD, RPM has been promoted as a transformative tool. The potential of RPM includes its ability to facilitate telehealth, reduce hospitalizations, reduced patient documentation, improve clinical team efficiency and workflow, enhance patient independence, promote the use of PD and improve the patient experience by reducing anxiety and improving comfort with PD, and potentially improve patient survival.^2^ Barriers include cost, data privacy, technological literacy, and challenges in patient connectivity. Currently, information collected from connected automated APD cyclers can be distilled into three key domains: (*1*) monitoring treatment adherence, (*2*) monitoring PD catheter function and mechanical flow issues, and (*3*) assessing daily fluid removal to assist with volume management. However, current evidence reveals a gap between this theoretical potential and proven clinical outcomes, particularly in preventing transitions from PD.

A significant challenge in PD remains the failure to address infections, particularly peritonitis, which continues to be the leading cause of PD dropout to hemodialysis.^3^ Early detection of peritonitis using a remote monitoring platform could potentially improve treatment timeliness and peritonitis-related outcomes. Notably, nearly one in five peritonitis episodes results in catheter removal and PD termination.^4^ The Relationship Between Presentation and the Time of Initial Administration of Antibiotics With Outcomes of Peritonitis in Peritoneal Dialysis Patients (PROMPT) study, which evaluated the timing of antibiotic administration after presentation for peritonitis, found that each hour of delay was associated with a 5.5% increased risk of peritonitis treatment failure.^5^ Current technologies, including inline sensors that detect early signs of peritonitis such as effluent turbidity, show promise. These devices are largely currently restricted to APD not continuous ambulatory PD (CAPD), which is the predominant dialysis modality in many countries outside the United States and have not yet been integrated into routine clinical practice.^6,7^ In this context, devices such as CloudCath have demonstrated encouraging preliminary results for the early identification of peritonitis.^7,8^ Whether these advances will translate into reduced morbidity and lower rates of transition to hemodialysis requires confirmation in further clinical trials.

Observational studies, such as those by Corzo *et al.* and Chaudhuri *et al.*, suggest that remote monitoring platforms may reduce transitions from PD to hemodialysis.^9,10^ Corzo *et al.* conducted a retrospective analysis of 558 patients on PD in Colombia and, using propensity matching, reported lower rates of hemodialysis transition among those using RPM (0.08 versus 0.18 episodes per patient-year).^9^ However, the study has notable limitations, including unclear patient selection criteria, no published protocols regarding how RPM data were used, and hemodialysis transition rates lower than typically observed in US cohorts.^9^ In addition, it remains unclear how common causes of hemodialysis transition reported by them—such as peritonitis, intra-abdominal pathology, or inadequate clearance—were identified and acted on through RPM. Without clarity on what interventions were triggered in response to flagged issues, the findings raise important questions regarding causality.

Similarly, the study by Chaudhuri *et al.* compared patients using a remote treatment monitoring platform with those not using it.^10^ This platform relied on patients engaging with the platform to provide treatment details, vital signs, and complications.^10^ Among the 6343 patients who registered online for remote treatment monitoring, 65% were nonusers and 25% were frequent users (entered >15 treatments). Frequent users had a lower risk of transitioning to hemodialysis, but compared with nonusers, the median extension on PD was only 13 days.^10^ The system's dependence on patient-reported data, absence of reporting on the causes of hemodialysis transition, and the shorter PD vintage among frequent users all highlight potential confounding factors, including differences in patient engagement and technology comfort that may account for the observed associations.^10^

**Figure 1:**
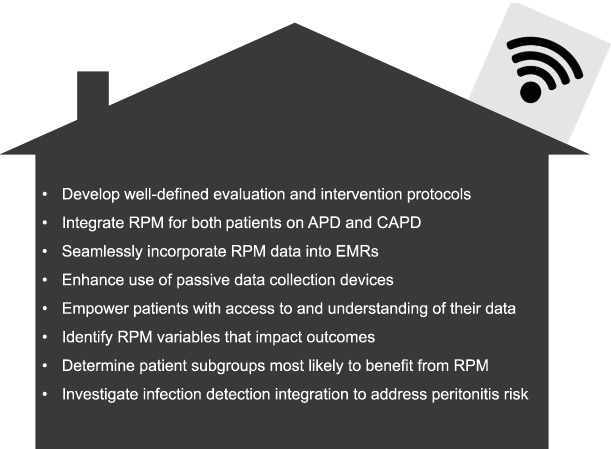
**Future needs for RPM in PD.** APD, automated peritoneal dialysis; CAPD, continuous ambulatory PD; EMR, electronic medical records; PD, peritoneal dialysis; RPM, remote patient monitoring.

By contrast, randomized trials provide more compelling evidence against RPM as an effective strategy to extend PD duration.^11,12^ The Paniagua *et al.* trial randomized 403 patients to remote monitoring APD and 398 to standard APD without monitoring, assessing a primary composite outcome of all-cause mortality, first adverse event, and hospitalization.^12^ The study found no difference in this primary outcome and no significant difference in hemodialysis transition rates. The study found more all-cause and cardiovascular deaths in the non-RPM group. Therefore, one could argue that the increased survival in the RPM group may have allowed patients to remain on PD longer. However, the increased risk of death in the non-RPM group, primarily driven by cardiovascular mortality that was observed was in the context of slightly higher age, lower levels of residual urine volume, and higher baseline cardiovascular risk factors in the non-RPM group (*i.e*., all ten patients with peripheral vascular disease the study were in the non-RPM group).^12^ Similarly, the Assessment of Telehome Monitoring in Patients on Peritoneal Dialysis: A Multicentre Randomized Controlled Trial (CONNECT) Trial, a robust multicenter randomized controlled trial enrolling across 11 sites and 467 patients allowing both patients on CAPD and APD in Canada using a comprehensive telecare platform including electronic clinical data entry, updated health information for the clinical team, secure text, picture, and video messaging. The study found no significant difference in hemodialysis transition rates between RPM and standard care.^11^ Collectively, these trials consistently demonstrate that, as currently implemented, RPM does not meaningfully delay transition to hemodialysis.^11,12^

For RPM to achieve its potential, it must move beyond passive data collection. Integration with electronic medical records, laboratory data, comorbidity profiles, and infection alerts is essential. Emerging artificial intelligence–driven tools may enhance the ability to contextualize and analyze RPM data, identifying subtle trends before clinical deterioration occurs.^13^ However, the effectiveness of RPM depends on structured workflows, standardized protocols, and timely clinical responses.

Owning a treadmill does not improve cardiovascular health without use; similarly, RPM without structured interpretation and actionable response plans does not extend PD therapy duration. Data must translate into protocolized and timely interventions, patient education, and system-level action. Observational studies and randomized controlled trials may have tested the presence of the treadmill but need to evaluate its structured and ongoing use. Perhaps RPM could be better enhanced with dedicated teams devoted to remote monitoring outside of the routine clinical care team such as monitoring centers routinely employed by home alarm system companies. Notably, a recent survey found that only 31% of clinicians report having standardized protocols for incorporating RPM data into clinical practice, underscoring the variability in its current implementation and lack of standardized integration into clinical workflows.^14^

In summary, although RPM in PD remains a promising tool for potential outcome improvement, current evidence does not support its consistent effectiveness in extending PD technique survival by preventing hemodialysis transitions. High-quality trials, artificial intelligence-enhanced analytic systems, systematic methods of monitoring, electronic medical records integration, and infection-focused monitoring approaches will be essential to realize the full potential of RPM in the future (Figure 1). It is important that as RPM is implemented on a wide scale that it narrows but not widen disparities relating to digital literacy and Internet access which may favor its use among certain groups of patients. Issues relating to privacy and data security may also be underappreciated barriers to adoption. The value of RPM must move beyond connected cyclers and include patients on CAPD. At present, the evidence for extending technique survival *via* RPM remains limited, although this may change with advancements in implementation and technology.

## References

[1] Manera KE Johnson DW Craig JC, .; SONG-PD Workshop Investigators. Establishing a core outcome set for peritoneal dialysis: report of the SONG-PD (Standardized outcomes in nephrology-peritoneal dialysis) consensus workshop. Am J Kidney Dis. 2020;75(3):404–412. doi:10.1053/j.ajkd.2019.09.017 31955922

[2] Talbot B Farnbach S Tong A, . Patient and clinician perspectives on the use of remote patient monitoring in peritoneal dialysis. Can J Kidney Health Dis. 2022;9:20543581221084499. doi:10.1177/20543581221084499 35340772 PMC8941702

[3] Lambie M Zhao J McCullough K, .; PDOPPS Steering Committee. Variation in peritoneal dialysis time on therapy by country: results from the peritoneal dialysis outcomes and practice patterns study. Clin J Am Soc Nephrol. 2022;17(6):861–871. doi:10.2215/CJN.16341221 35641246 PMC9269666

[4] Al Sahlawi M Zhao J McCullough K, . Variation in peritoneal dialysis-related peritonitis outcomes in the peritoneal dialysis outcomes and practice patterns study (PDOPPS). Am J Kidney Dis. 2022;79(1):45–55.e1. doi:10.1053/j.ajkd.2021.03.022 34052357

[5] Muthucumarana K Howson P Crawford D Burrows S Swaminathan R Irish A. The relationship between presentation and the time of initial administration of antibiotics with outcomes of peritonitis in peritoneal dialysis patients: the PROMPT study. Kidney Int Rep. 2016;1(2):65–72. doi:10.1016/j.ekir.2016.05.003 29142915 PMC5678844

[6] Jain AK Blake P Cordy P Garg AX. Global trends in rates of peritoneal dialysis. J Am Soc Nephrol. 2012;23(3):533–544. doi:10.1681/ASN.2011060607 22302194 PMC3294313

[7] Mehrotra R Williamson DE Betts CR, . A prospective clinical study to EvaluAte the AbiliTy of the CloudCath system to detect peritonitis during In-Home peritoneal dialysis (CATCH). Kidney Int Rep. 2024;9(4):929–940. doi:10.1016/j.ekir.2024.01.033 38765568 PMC11101817

[8] Briggs B Garcia-Garcia G Ibarra-Hernandez M, . Performance characteristics of a prototype dialysate turbidity monitoring system to detect peritonitis in patients receiving peritoneal dialysis. Perit Dial Int. 2024;44(6):419–425. doi:10.1177/08968608231195532 37723968

[9] Corzo L Wilkie M Vesga JI, . Technique failure in remote patient monitoring program in patients undergoing automated peritoneal dialysis: a retrospective cohort study. Perit Dial Int. 2022;42(3):288–296. doi:10.1177/0896860820982223 33380265

[10] Chaudhuri S Han H Muchiutti C, . Remote treatment monitoring on hospitalization and technique failure rates in peritoneal dialysis patients. Kidney360. 2020;1(3):191–202. doi:10.34067/KID.0000302019 35368632 PMC8809254

[11] Jain AK. Telemonitoring and the CONNECT Trial *ISPD* Dubai, UAE; 2024.

[12] Paniagua R Ramos A Avila M, .; Mexican Nephrology Collaborative Study Group. Remote monitoring of automated peritoneal dialysis reduces mortality, adverse events and hospitalizations: a cluster-randomized controlled trial. Nephrol Dial Transplant. 2025;40(3):588–597. doi:10.1093/ndt/gfae188 39165115 PMC11997789

[13] Kotanko P Zhang H Wang Y. Artificial intelligence and machine learning in dialysis: ready for prime time? Clin J Am Soc Nephrol. 2023;18(6):803–805. doi:10.2215/CJN.0000000000000089 36795031 PMC10278848

[14] El Shamy O Fadel R Weinhandl ED, . Variations in provider practices in remote patient monitoring on peritoneal dialysis in the USA and Canada. Perit Dial Int. 2025;45(3):190–194. doi:10.1177/08968608241270294 39105257

